# Understanding primary care physicians’ perceptions of large language model adoption in clinical practice: A qualitative research protocol leveraging the technology adoption behavior framework

**DOI:** 10.1371/journal.pone.0355491

**Published:** 2026-08-07

**Authors:** Benny Bikash Pokharel, Monica Hsu, Lindsay Hedden, Laura Nimmon, David E. Bloom, Sian H. Tsuei

**Affiliations:** 1 Faculty of Medicine, University of British Columbia, Vancouver, British Columbia, Canada; 2 Faculty of Health Sciences, Simon Fraser University, Vancouver, British Columbia, Canada; 3 Department of Global Health and Population, Harvard T.H. Chan School of Public Health, Boston, Massachusetts, United States of America; University of Florida, UNITED STATES OF AMERICA

## Abstract

**Introduction:**

Large language models’ (LLMs’) rapid evolution and intersection with diverse groups and institutions require up-to-date policies, practices, and behaviors to ensure safe and effective implementation. Because physicians play a central role in care provision and face the dual mandate of embracing innovations and safeguarding patient welfare, understanding physicians’ views on LLMs can elucidate the complex interplay of technical, ethical, and professional considerations influencing LLM adoption.

**Methods and analysis:**

This qualitative study aims to employ a descriptive qualitative design to explore how primary care physicians perceive the adoption of LLMs in the context of their clinical practice. We plan to use semi-structured interviews with purposively sampled primary care physicians from British Columbia, Canada. The data collection will draw on the technology adoption behavior framework, a novel model that integrates the most advanced theories of technological uptake. We expect to use thematic analysis drawing on deductive and inductive approaches to describe physicians’ perceptions. The multidisciplinary research team will prepare and conduct reflexive memos and discussions to ensure nuanced interpretations.

**Dissemination:**

We aim to disseminate the findings through peer-reviewed journals, professional organizations, and policymaker briefings to support the development of policies, practices, and behaviors that support safe and effective LLM integration into health care.

**Strengths and limitations:**

- The study may provide timely input into relevant policies, practices, and behaviors for policymakers, health professionals, and patients around the use of large language models in health care services.
- This study uses the technology adoption behavior framework to guide the design of data collection and analysis.
- The semi-structured interview may reveal the interviewees’ internal views but limits the variety of insights gleaned.

## 1 Introduction

Developing appropriate policies, practices, and behaviors for new technologies is crucial for protecting and improving health care system performance. However, identifying the types and magnitude of relevant consequences can be challenging in the early stages of technological development and implementation. For example, electronic medical records were meant to provide high-quality documentation but were also associated with disrupted clinical workflow and increased provider burnout [1,2].

Designing policies that mitigate the harms and maximize the benefits from modern large language models (LLMs) can be similarly challenging. LLMs are a class of artificial intelligence systems trained on vast text datasets to understand and generate human-like language. In healthcare, their ability to process unstructured clinical information positions them as emerging tools for documentation, decision support, patient communication, and administrative workflows. Their wide-ranging capacity can help support clerical tasks, such as summarizing patient visits and drafting letters to patients [3]. They can also support diagnostic and therapeutic tasks, such as taking a patient’s history, interpreting certain investigations, recommending potential diagnoses, and suggesting investigations and therapies. In some cases, LLMs’ diagnostic performance may at times rival that of some human specialists [4–7]. These studies suggest promising performance in many contexts; however, this research is preliminary and subject to important limitations given the novelty of the field. Additionally, LLMs can also perpetuate biases, generate false information, and potentially displace humans from their jobs [8,9]. LLMs’ rapid development and intersection with multiple spheres of behavioral norms further complicate their assessment [10–13].

Studying how physicians consider the diverse benefits and risks can be particularly enlightening. First, their roles cross both leadership and frontline service provision. Second, they have to weigh the need to introduce advanced technologies for patient benefit against their potential harms [14,15]. Lastly, physicians using LLMs can improve their service quality and efficiency, but excessive reliance on LLMs can undermine physicians’ skills and long-term importance in the health care service market.

To reveal deeper insights into the complex multidimensional dynamics [16], this study thus aims to qualitatively examine *how physicians perceive the uptake of LLMs*. Overall, this study provides two contributions. First, this informs literature on how professions absorb new technologies amid evolving practical, legal, ethical, and existential considerations. Few technologies have had such wide implications. Second, clarifying how physicians balance the various considerations can grant policymakers the necessary insight to consider whether and how to adjust policies for optimal health system performance. Although a small but growing body of work has begun to examine clinicians’ perceptions of LLMs [17–19], prior studies are largely atheoretical or lightly theory informed and rarely focus on primary care. To our knowledge, no study in primary care has used an explicit framework to guide both data collection and analysis.

We focus on family physicians in Canada for two reasons. First, strong primary care can improve the quality, access, efficiency, and mortality [20–22]. Family physicians constitute the largest share of the physician workforce in this country [23], often gatekeeping patients’ access to other health care services. However, the estimated number of patients without a family doctor rose from 4.5 million in 2019 to about 6.5 million in 2023 [24], driving up public discontent and missed opportunities to improve health [25,26]. Understanding the motivations and reservations facing this group of health care service providers may thus be particularly helpful in unlocking the relevant support for downstream health benefits. Second, many family medicine diagnoses rely on verbal information and cognitive considerations, which coincide with LLMs’ support capacity. This may be particularly helpful in illustrating the physicians’ intricate affinity and tensions with such a technology.

## 2 Methodology

### 2.1 Overall study design

**Fig 1:**
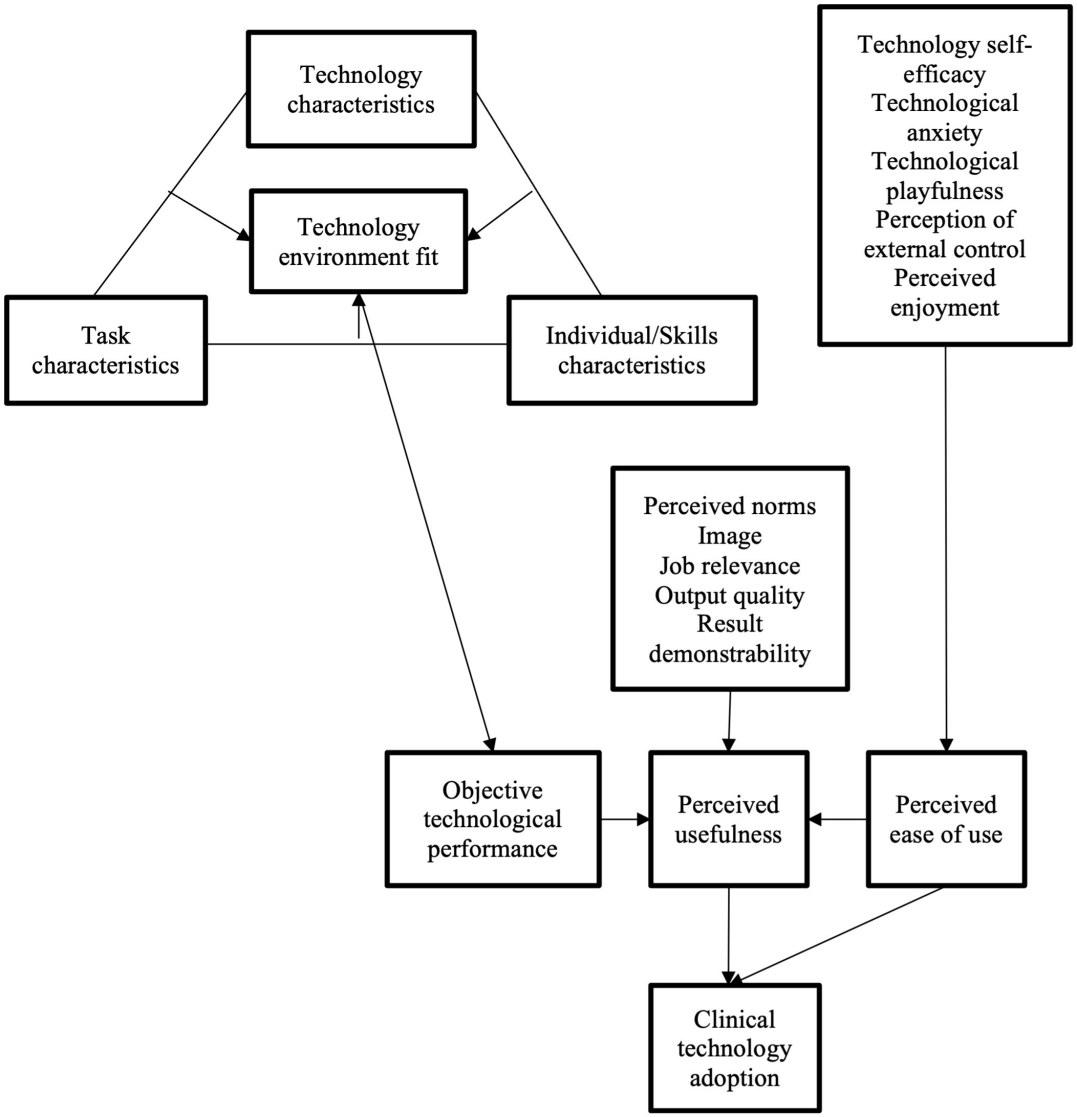
The expanded technology adoption behavior prediction framework.

We draw on the qualitative descriptive approach to capture the complexity of how physicians perceive the use of LLM tools [27,28]. This approach offers a “summary of events in the everyday terms of those events” with flexible sampling, data collection, analysis, and representation [29]. We will employ semi-structured interviews, which provides an in-depth understanding of participant perspectives [30]. We will use purposive sampling to ensure representation from those who use and avoid LLMs. The data collection and analysis will leverage the technological adoption behavior (TAB) framework. This is an expanded theoretical framework on technological uptake we developed to unite multiple evidence-based theoretical streams (Fig 1). The final analysis will conduct thematic analysis with deductive and inductive coding.

### 2.2 Theoretical framework

We followed the guidance from Varpio et al. in creating “a logically developed and connected set of concepts and premises—developed from one or more theories—that …scaffold a study” [31]. Fig 1 shows the TAB framework, and Appendix A (S1 Appendix) describes the historical evolution of the various frameworks underlying its development. The TAB framework builds on two established traditions in research on technology uptake: one emphasizing the role of user perceptions and the other emphasizing the fit between technology, tasks, and users. To make these links transparent, we organize this section into three parts. Section 2.2.1 outlines the importance of perceptions, drawing from models such as the Technology Acceptance Model (TAM) [32]. Section 2.2.2 describes the technology–environment fit tradition, including both early formulations [33,34] and more recent refinements [35]. Section 2.2.3 explains how TAB integrates these perspectives into a unified framework. Although some foundational contributions are older, they remain the basis for contemporary refinements, and we complement them with recent extensions [35] to ensure the framework reflects current scholarship.

#### 2.2.1 Importance of perception.

The former camp emphasizes that the motivation to incorporate a tool into users’ work stems from their perceptions. The two key perceptions are the perceived *ease of use* (i.e., the degree of expected difficulty in taking on a new technology) and perceived *usefulness* (i.e., the degree a technology is expected to improve users’ actions). Multiple individual, task, and social characteristics in turn affect these two factors.

The users’ perceived capacity to operate the technology effectively (i.e., self-efficacy and anxiety related to the technology) primarily influence the perceived *ease of use*. If the users perceive the technology to be playful, then learning to work with the technology becomes a spontaneous exploration. Furthermore, supportive resources for using the technology may also lower the perceived challenge [32].

In contrast, both technical and social considerations influence perceived *usefulness*. The technical considerations largely capture whether the technology can support the tasks’ needs. Higher technology’s relevance and expected output quality for the task may lead to higher apparent usefulness. The social considerations suggest that as more people use a technology, the normative pressure to use it also increases, potentially translating to a better image for the users [32].

#### 2.2.2 Importance of technology’s fit with the environment.

The latter camp emphasizes that research on adoption should instead focus on the technologies’ fit with the environment. The most advanced form includes the technology’s fit with the task and the user [33–35]. The task characteristics include the difficulty, routineness, complexity, and variety of tasks. For instance, as the tasks’ difficulty, complexity, and variety increase, the technologies may need to be more sophisticated and adaptable to be useful. This may then improve the chances of incorporating the technology into the workflow. The user’s characteristics includes the person’s education, training, experience, and motivation. For example, less educated users may require an adjustment of the technology’s user interface for better uptake.

#### 2.2.3 Unification of the two camps.

The early scholars who studied technology-environment fit recognized that fit alone was insufficient; the fit needs to be recognized as useful to affect technological adoption [34]. However, the theoretical expansion of this vein has subsequently focused on the interdependence of the technology, user, and tasks. To capture the potential factors affecting LLM uptake more comprehensively, we combine the two veins of theoretical development together into the TAB framework.

The TAB framework follows the former camp’s tradition by suggesting that both perceived *ease of use* and perceived *usefulness* drive technological adoption. It also incorporates the latter camp’s emphasis on the technology’s fit with the user and task. We follow Goodhue and Thompson’s conceptualization to integrate the two camps by suggesting that the technology-environment fit feeds into the perceived usefulness. We add in additional individual and social considerations from other empirical literature.

### 2.3 Study setting, sample, and recruitment

We focus on the Canadian province of British Columbia. Despite the government’s encouragement for health care providers in this region to use innovative health care technologies, physicians’ uptake of digital technologies may be delayed and variable [36–38].

We will purposively sample practicing physicians with varying levels of LLM usage, ranging from frequent, occasional, and nearly no use (i.e., the physician has used LLMs 50 + , 5–50, and <5 times in their lifetime, respectively, in the context of health care services). The sampling aims to reach sufficiency, achieved when there is enough evidence and depth of conceptual understanding to meaningfully answer the research question [39]. We will also ensure variability in terms of the years of practice, which may affect participants’ perceptions of digital technologies. The diverse perspectives may help reveal broadly applicable insights and support analytical generalizability [40]. We will exclude family physicians who work exclusively in non-clinical roles (e.g., academic, research, or administrative). We aim to recruit participants from existing professional networks and local physician organizations such as Doctors of British Columbia, BC Family Doctors, and British Columbia College of Family Physicians. Recruitment will occur through organizational listserv emails, posted invitations on member websites, and outreach at educational events. In addition, physicians known to the research team may be approached via personal contacts; snowball sampling will be used when participants recommend colleagues.

### 2.4 Semi-structured interview and question guide

The semi-structured approach of the interview allows for depth and flexibility as we explore the central phenomenon of interest. We designed the interview guide to start with open-ended questions around key constructs of the TAB framework (Table 1). The current version is designed to be more comprehensive, and the first two interviews will serve as pilots to ensure smooth interview flow. The guide includes prompts to allow further exploration of key predefined concepts in the TAB framework. Because different experiences and perspectives in the application of LLMs may drive varying perceptions of LLM usage, interviewers will briefly clarify the contexts under which the participants have interacted with LLMs. The interviewers will do so at the outset of each interview. The questions will ask the participant to clarify the examples of LLMs used or considered, intended purpose of such LLMs (e.g., prevention, diagnostics, or therapy), target user groups, potential hardware required, and relevant settings of use. Additionally, we will prompt for disconfirming evidence to enrich the data collection process [41], actively probing for accounts that contradict or challenge emerging patterns or assumptions.

**Table 1:** Semi-structured interview guide.

Question	TAB Construct	Associated Variable
In what context have you used, or do you imagine using, LLMs in your practice? When you think about LLMs in your answers today… a) What specific examples or types of tools are you primarily thinking of? B) are they used for diagnostics, prevention, or treatment? C) Who do you see as the main user of these tools? D) At a broad level, under what settings have you used or imagine using these tools in clinical practice? E) What types of hardware do you anticipate including (e.g., computer or smartphone)?		
What do large language models (LLMs) mean to you?	TEF/PEU/PU	
• If applicable, how do you foresee using LLMs?	TEF	Task-Tech Relationship
• What tasks do you foresee using LLMs for?	TEF	Task-Tech Relationship
• How do you plan to expand your own skills to better use LLMs?	TEF	Individual-Tech Relationship
Can you share some examples of how you have used LLMs? Did you enjoy or dislike those experiences? Why?	PEU	
What do you like or dislike about them?	PEU	
• In terms of general use? Medical use?	PEU	Tech Enjoyment
• How difficult is it to generate prompts?	PEU	Tech Self-Efficacy
• How has it affected your sense of autonomy as a professional?	PEU	Tech Enjoyment
What would help you feel more comfortable with using it?	PEU	
• Do you feel that there are resources to help you learn to work with LLMs?	PEU	Perception of External Control
How do you see LLMs affecting your role as a physician?	PU	Image
• With regards to the medical community? Patients? Society at large?	PU	Subjective Norm
How do you think doctors who use LLMs as a primary component of their patient care would be viewed?	PU	Image Subjective Norm
• By the medical community? Patients? Society at large?	PU	Subjective Norm Image
• How do you think the widespread adoption of LLMs might affect job security or career prospects for physicians?	PU	Subjective Norm
What worries you about LLM use in medicine?	PEU/PU	Tech Anxiety
• For medical doctors? For patients?	PEU/PU	Tech Anxiety Output Quality
• Any concrete results that you expect?	PU	Results Demonstrability
How do you perceive the learning curve for integrating LLMs?	PEU	Tech Anxiety
What excites you most about LLM use in medicine?	PEU/PU	
• For medical doctors? For patients?	PEU/PU	
• Any concrete results that you expect?	PU	Results Demonstrability
Compared with other professions, are there any differences in how LLMs affect doctors? How so? Why or why not?	PU	Job Relevance
How do you see LLMs affecting patients?	PU	Output Quality
• What tangible, observable, or communicable benefits do you foresee?	PU	Results Demonstrability
• For patients? Medical doctors? Medical community?	PU	Results Demonstrability
• What tangible, observable, or communicable drawbacks do you foresee?	PU	Results Demonstrability
• For patients? Medical doctors? Medical community?	PU	Results Demonstrability

Note: “TAB Construct” refers to the three overarching domains of the Technology Adoption Behavior framework (technology–environment fit [TEF], perceived ease of use [PEU], and perceived usefulness [PU]). “Associated Variable” refers to the specific theory-derived dimensions within each construct that informed the design of interview questions. This structure illustrates how each question is explicitly linked to the TAB framework..

One primary researcher will conduct the interviews using in-person, telephone, or video conferencing approaches. All the interviews will be recorded with a physical recording device or video conferencing software and subsequently transcribed verbatim and deidentified.

### 2.5 Data analysis

The diverse background of our team members helps ensure that the data interpretation captures diverse perspectives and interpretations. They include a medical trainee, a practicing family physician, two medical educators, an economist, and three health system researchers. The team will write reflexive memos and engage in reflexive dialogues on how their identity may have influenced their data interpretation. The senior author will be attuned to all team members perspectives, carefully accounting for all contributions [42,43].

We aim to use both deductive and inductive coding approaches to flexibly understand and capture the content from the participants. Table 2 shows the series of deductive codes based on the TAB framework. The inductive coding refines the existing codes and / or develop new ones based on the empirically collected data [44]. We will rely on this approach when the data extend beyond the existing codes developed based on the TAB framework. Additionally, we will write analytical memos throughout the coding process to document how the empirical findings may extend the TAB framework and refine relevant codes appropriately. Primary coding will be conducted by a new member of the research team after the person is trained in the TAB framework and qualitative coding. The person will be in charge of developing additional codes, and every code addition, removal, and refinement will be discussed with the senior author. The adjustments will be discussed with the rest of the team every month until the codebook is finalized. The team will aim to balance the TAB framework with the team’s diverse perspectives to focus analysis [42].

**Table 2:** Deductive codebook.

Code	Definition	Category
Interventions: task level	Proposed interventions on task characteristics	TEF
Interventions: technology level	Proposed interventions on characteristics of a technology	TEF
Interventions: individual level	Proposed interventions to individuals using a technology	TEF
Fit between task and technology	Characteristics of a task and a technology that synergize	TEF
Fit between individual and technology	Characteristics of an individual or an individual’s skills and a technology that synergize	TEF
Fit between task and individual	Characteristics of an individual or an individual’s skills and a task that synergize in the context of technological use	TEF
Technology self-efficacy	Degree to which an individual believes that they can perform the task using the technology	PEU
Perception of external control	Degree to which one believes that resources exist to support the use of the system.	PEU
Technological anxiety	Degree to which one is apprehensive or fearful of using the technology	PEU
Technological playfulness	Degree of cognitive spontaneity in interaction with the technology	PEU
Technological enjoyment	Degree to which using a technology is enjoyable in its own right aside from completing the actual task	PEU
Objective usability	Degree to which a technology makes completion of a task easier	PEU
Image	Degree to which a user perceives using the technology will enhance their status in their social system	PU
Job relevance	Degree to which a user perceives that the technology is relevant to their job	PU
Output quality	Degree to which a user believes that the technology adequately performs tasks	PU
Result demonstrability	Degree to which a user perceives that results of using a technology are tangible, observable, or communicable	PU
Subjective norm	Degree to which a user perceives that people that are important to them believe they should use a technology	PU

We will iteratively discuss within the group to distill the meaning of the findings, and as the relationships and dynamics of the codes emerge, this will lead us towards broader themes [45]. We aim to generate clear and concise definitions for each theme and review and refine the identified themes to ensure accurate representation. We will use NVivo to conduct all analysis.

### 2.6 Patient and public engagement

Although the team did not consult patients or the public in developing the study, the work is designed to inform the ongoing need for meaningful policies that can protect the safety and quality of care when introducing AI tools [46].

### 2.7 Ethical considerations

We have obtained approval from the harmonized ethics review board (UBC Behavioral Research Ethics Board; H25-01658). All potential participants will first receive recruitment materials approved by the review board, and those who agree to participate will be given informed consent forms also approved by the board. At the start of the interview, we will inform the participants of the study’s aims, the participants’ rights (including the right to withdraw at any point without repercussions), sharing of deidentified data, and potential resources for post-interview debriefing. After the transcriptions, the original audio files will be destroyed and the transcripts will be deidentified. The team will work only with the deidentified version of the data.

### 2.8. Study status

We have only completed the study design, and we have not yet begun participant recruitment or data collection. We expect participant recruitment and data collection to be completed within six months (anticipated timeline from August 1, 2025, to January 31, 2026). We expect that once the data collection begins, we can complete the study and produce results within a year.

## 3 Discussion

### 3.1 Dissemination

We will share the findings with local, provincial, and national physician groups, policymakers, and health care organization leaders. We will also present the work at conferences and publish the findings in a journal.

## Supporting information

S1 Appendix Review of technology uptake frameworks leading up to the technological adoption behavior framework. (DOCX)

S1 Fig Illustration highlighting key differences between (A) the theory of reasoned action (TRA) and (B) the theory of planned behavior (TPB). TRA assumes that the intention to complete a behavior is sufficient for actual behavior. TPB adds perceived behavioral control as another variable contributing to behavioral intention and as a variable that independently contributes to actual behavior.
(TIF)

S2 Fig The technology acceptance model (TAM) combined with TPB. The contribution of TAM is colored in blue. Both these variables—perceived usefulness and perceived ease of use—specifically refer to features of a technology that ultimately contribute to users’ uptake of the technology.
(TIF)

S3 Fig Simplified TAM3 model that combines TAM with TRA/TPB to develop a technological use behavior prediction model. *External variables include perceived ease of use, subjective norm, image, and result demonstrability. **External variables include computer self-efficacy, computer anxiety, and computer playfulness, and perceptions of external control.
(TIF)

S4 Fig Goodhue and Thompson’s task technology fit framework of technological adoption relative to TAM3. The interplay between task/technology and individual/technology is key for appropriate task technology fit, which is a predictor of technological performance. User evaluation is sufficient in measuring technological performance. This figure also demonstrates that technological performance is an external variable that influences the perceived usefulness variable in TAM, ultimately influencing technological use behavior.
(TIF)

S5 Fig Fit among individuals, task, and technology (FITT) framework of technological adoption. FITT emphasizes the connection between task and individual as another key benchmark for appropriate technological fit while the TTF framework does not.
(TIF)

S6 Fig Ruyobeza et al.’s fit among individuals, skills, tasks, and technology framework (2023). (TIF)
