# Supplementary material for: Understanding primary care physicians’ perceptions of large language model adoption in clinical practice: A qualitative research protocol leveraging the technology adoption behavior framework
Source: PLoS One. 2026 Aug 7;21(8):e0355491. doi: 10.1371/journal.pone.0355491 (PMC13450787; doi:10.1371/journal.pone.0355491)
Supplement: Supplementary file 1 [file pone.0355491.s001.docx]

# S1 Appendix. Review of technology uptake frameworks leading up to the technological adoption behavior framework

We review here the developments that led to the technological adoption behavior (TAB) framework. Section S1.1 highlights how scholars first decided to focus on the user’s perceptions as a driver of their behaviors. Section S1.2 expands on this further by suggesting that the key perception relevant to technology uptake relates to that of the technology’s *ease of use* and *usefulness*. Section S1.3 describes a divergent vein that began to focus on the technology’s fit with the environment (i.e., user and task). Section S1.4 describes the motivation to integrate the frameworks from sections S1.2 and S1.3 that drove the development of the TAB framework, and we briefly describe the TAB framework’s components.

## S1.1. Theories of reasoned action and planned behavior

Fishbein and Ajzen proposed the theory of reasoned action (TRA; Figure S1A), which attempted to identify the factors that contribute to an intention to perform certain actions [1]. This theory argues that a user’s intention to perform a behavior depends on the user’s attitude (i.e., the degree to which a behavior is evaluated as positive) and subjective norms (i.e., the degree to which someone perceives that other people want them to perform a behavior). Although this theory can predict a user’s behavioral motivation, it does not consistently translate to actual behaviors [2].

The disconnect led to the updated theory of planned behavior (TPB; Figure S1B). The additional category of “perceived behavioral control” captures the factors influencing the perceived difficulty of the behavior of interest. The perceived behavioral control should not only affect actual behavior but also the intention to perform a behavior.

**Figure S1. Illustration highlighting key differences between (A) the theory of reasoned action (TRA) and (B) the theory of planned behavior (TPB).** TRA assumes that the intention to complete a behavior is sufficient for actual behavior. TPB adds perceived behavioral control as another variable contributing to behavioral intention and as a variable that independently contributes to actual behavior.

## S1.2. Technology acceptance model

Davis then extended TPB into the technology acceptance model (TAM, Figure S2) by further theorizing about what drives a user’s attitude toward a technology. The TAM proposes that the *perceived usefulness* and *perceived ease of use* are key in adjusting users’ behavioral intentions [3]. Perceived usefulness pertains to the degree to which a user believes that a technology will improve their actions, and *perceived ease of use* is the degree to which a user believes that use of a technology will require minimal effort.

**Figure S2. The technology acceptance model (TAM) combined with TPB.** The contribution of TAM is colored in blue. Both these variables—perceived usefulness and perceived ease of use—specifically refer to features of a technology that ultimately contribute to users’ uptake of the technology.

Venkatesh and Bala further clarified the external variables that could affect both perceived ease of use and perceived usefulness in the most refined model, TAM3 (Venkatesh & Bala, 2008). Drawing on the context of computers, the most significant variables for predicting the perceived ease of use were computer self-efficacy, computer anxiety, computer playfulness, and perceptions of external control [4]. In contrast, the variables that were found to most contribute to perceived usefulness were perceived ease of use, subjective norm, image, and result demonstrability [4].

Furthermore, the TAM3 incorporated the TRA/TPB considerations into the TAM by adding subjective norms as an external variable that contributes to perceived usefulness (Figure S3).

**Figure S3. Simplified TAM3 model that combines TAM with TRA/TPB to develop a technological use behavior prediction model**. *External variables include perceived ease of use, subjective norm, image, and result demonstrability. **External variables include computer self-efficacy, computer anxiety, and computer playfulness, and perceptions of external control.

## S1.3. Task technology fit, FITT, and FISTT

### S1.3.1 Task technology fit

Although some experts use TAM3 to study technological adoption, others suggest that it is insufficient. The users’ proficiency with the technology and the technology’s ability to support a desired task are also key [5]. Figure S4 shows Goodhue and Thompson’s task technology fit (TTF) framework, which suggests that the degree to which a technology actually helps with a desired action is key for better technological adoption [6]. TTF argues that appropriate interplay among individual, technology, and task characteristics is crucial for maximizing technological performance, which in turn improves perceived usefulness.

**Figure S4. Goodhue and Thompson’s task technology fit framework of technological adoption relative to TAM3.** The interplay between task/technology and individual/technology is key for appropriate task technology fit, which is a predictor of technological performance. User evaluation is sufficient in measuring technological performance. This figure also demonstrates that technological performance is an external variable that influences the perceived usefulness variable in TAM, ultimately influencing technological use behavior.

### S1.3.2 FITT

Figure S5 shows that the fit among individuals, task, and technology (FITT) framework extends beyond the conventional TTF by suggesting that the factors affecting technological adoption are not mutually independent [7]. Rather, the interactions among the task, individual, and technology factors can influence technological adoption. Optimal fit is when individuals are sufficiently motivated and knowledgeable to execute a certain task, the technology is sufficiently functional to support a given task, and that the user is sufficiently trained to appropriately use a given technology.

**Figure S5. Fit among individuals, task, and technology (FITT) framework of technological adoption.** FITT emphasizes the connection between task and individual as another key benchmark for appropriate technological fit while the TTF framework does not.

### S1.3.3 FISTT

Ruyobeza et al.’s fit among individuals, skills, tasks, and technology (FISTT) framework argues that current and future skills are primarily responsible for the “individual characteristics” variable in the FITT framework [8]. The model suggests that “skills” could proxy many of the meaningful dimensions of the “individual” dimensions, but it retains the “individual” dimension to account for the motivation for task completion (Figure S6).

**Figure S6. Ruyobeza et al.’s fit among individuals, skills, tasks, and technology framework (2023).**

## S1.4. TAB framework

So far, the frameworks reviewed suggest that two major aspects—perceived *ease of use* and perceived *usefulness—*mainly affect technological adoption. The technology-environment fit is a second vein of research that originally highlighted the importance of perceived usefulness, but over time scholars increasingly focused on the characteristics of the fit, rather than reintegrating into the broader folds of the technology uptake frameworks.

We address this deficiency in TAB. We reintegrate the most advanced forms of the technology-environment fit framework (i.e., FITT/FISTT) as one of the antecedents supporting the perceived usefulness factor. This is in line with Goodhue and Thompson’s recognition that technology-environment fit affects technological uptake via perceived usefulness.

To ensure the usefulness of TAB, we also expanded the subsidiary input factors feeding into the perceived *ease of use* and perceived *usefulness*. Technology self-efficacy, technology anxiety, technology playfulness, perception of external control, perceived enjoyment, and objective usability influence perceived ease of use [4]. Subjective norms, image, job relevance, output quality, result demonstrability and perceived ease of use affect perceived usefulness [4], in addition to the technology’s fit with the user and task characteristics.

## S1.5. References

1.	Fishbein M, Ajzen I. Belief, Attitude, Intention, and Behavior: An Introduction to Theory and Research. Boston, MA: Addison-Wesley Pub. Co. Boston, MA.; 1975.

2.	Ajzen I. The theory of planned behavior. Organizational Behavior and Human Decision Processes. 1991;50: 179–211. doi:10.1016/0749-5978(91)90020-T

3.	Davis FD. Perceived usefulness, perceived ease of use, and user acceptance of information technology. MIS Quarterly. 1989;13: 319. doi:10.2307/249008

4.	Venkatesh V, Bala H. Technology acceptance model 3 and a research agenda on Interventions. Decision Sciences. 2008;39: 273–315. doi:10.1111/j.1540-5915.2008.00192.x

5.	Dixon DR. The behavioral side of information technology. Int J Med Inform. 1999;56: 117–123. doi:10.1016/s1386-5056(99)00037-4

6.	Goodhue DL, Thompson RL. Task-technology fit and individual performance. MIS Quarterly. 1995;19: 213–236. doi:10.2307/249689

7.	Ammenwerth E, Iller C, Mahler C. IT-adoption and the interaction of task, technology and individuals: a fit framework and a case study. BMC Med Inform Decis Mak. 2006;6: 3. doi:10.1186/1472-6947-6-3

8.	Ruyobeza BB, Grobbelaar SSS, Botha A. From FITT to FISTT: The task-skills fit before the introduction of assistive, digital health technologies. Heliyon. 2023;9: e16885. doi:10.1016/j.heliyon.2023.e16885
