# Supplementary material for: Understanding primary care physicians’ perceptions of large language model adoption in clinical practice: A qualitative research protocol leveraging the technology adoption behavior framework
Source: PLoS One. 2026 Aug 7;21(8):e0355491. doi: 10.1371/journal.pone.0355491 (PMC13450787; doi:10.1371/journal.pone.0355491)
Supplement: Supplementary file 2 [file pone.0355491.s009.docx]

**Editor’s comments**

1. Please ensure that your manuscript meets PLOS ONE's style requirements, including those for file naming. The PLOS ONE style templates can be found at

<https://journals.plos.org/plosone/s/file?id=wjVg/PLOSOne_formatting_sample_main_body.pdf> and

[https://journals.plos.org/plosone/s/file?id=ba62/PLOSOne_formatting_sample_title_authors_affiliations.pdf](https://track.editorialmanager.com/CL0/https:%2F%2Fjournals.plos.org%2Fplosone%2Fs%2Ffile%3Fid=ba62%2FPLOSOne_formatting_sample_title_authors_affiliations.pdf/1/010f019912de6db9-01ace8e4-b1a5-4f19-b75d-29d0ea5c4f29-000000/_0R8Dd2EBSqpt0RCSTwDIGBLc0CBuf1U-GEpbzPK3dQ=227)

**Our team reviewed this again. To our knowledge, our format aligns with PLOS ONE’s style requirements.**

2. Please note that PLOS One has specific guidelines on code sharing for submissions in which author-generated code underpins the findings in the manuscript. In these cases, we expect all author-generated code to be made available without restrictions upon publication of the work. Please review our guidelines at <https://journals.plos.org/plosone/s/materials-and-software-sharing#loc-sharing-code> and ensure that your code is shared in a way that follows best practice and facilitates reproducibility and reuse.

**This piece seems to refer to computational code instead of qualitative codes. We do not expect to use any computational code in our study, and we have explicitly described the deductive codes that we plan to use.**

3. Thank you for stating the following in the Competing Interests section:
“Dr. Sian Tsuei serves as a member on the Artificial Intelligence Advisory Council for the College of Canadian Family Physicians.”

Please confirm that this does not alter your adherence to all PLOS ONE policies on sharing data and materials, by including the following statement: "This does not alter our adherence to PLOS ONE policies on sharing data and materials.” (as detailed online in our guide for authors http://journals.plos.org/plosone/s/competing-interests). If there are restrictions on sharing of data and/or materials, please state these. Please note that we cannot proceed with consideration of your article until this information has been declared.

**We have now included in our manuscript the statement that "[t]his does not alter our adherence to PLOS ONE policies on sharing data and materials.”**

4. Your ethics statement should only appear in the Methods section of your manuscript. If your ethics statement is written in any section besides the Methods, please delete it from any other section.

**We have included the ethics statement in only the Methods. We removed the ethics statement from the Abstract section.**

5. If the reviewer comments include a recommendation to cite specific previously published works, please review and evaluate these publications to determine whether they are relevant and should be cited. There is no requirement to cite these works unless the editor has indicated otherwise.

**Thank you. The reviewers have not specifically asked us to cite any previously published work.**

**Reviewer 1 Comments**

Dear Authors,

This study addresses a relevant and timely topic using a sound methodology. The manuscript is well written and thoughtfully prepared. Although I usually aim to provide critical and constructive feedback, I find this study protocol to be excellent. I have no concerns or further comments. I look forward to seeing the results of this study.

**Thank you for the kind comment. We appreciate that the work was of interest to you and met your standard. Please do look out for our subsequent work. We hope that it’llcontinue to be of interest.**

**Reviewer 2 Comments:**

1. A summary of what previous studies found out on primary care physicians´ (or health professionals´) perspectives on large language models is missing. I agree that primary care physicians are those who do the heavy lifting, so understanding their perspectives is especially important. However, some studies, quantitative and qualitative, must exist out there, so it would be interesting to contextualize your study idea in the current scholarly discussion/state of the art.

**While previous work has examined general healthcare workers’ perception of LLMs [1], to our knowledge, assessment of clinician/healthcare worker perception of LLMs guided by theory is not present in the literature. The relevant sections of the text reads:**

**Although a small but growing body of work has begun to examine clinicians’ perceptions of LLMs [17–19], prior studies are largely atheoretical or lightly theory informed and rarely focus on primary care. To our knowledge, no study in primary care has used an explicit framework to guide both data collection and analysis.**

2. Para 2.2. is hard to follow. There is hardly any reference in the text but you seem to make a summary of what “experts” (who are they?) belive and did, you mention “camps” (what is that exactly?) without referencing any of your claims. References n. 28, 29, 30 are 17, 19 and 30 years old respectively, pretty outdated for a research protocol on LLMs.

It seems that the theoretical framework is your work, developed from previous studies that should be explained and referenced. Part of that is now in the appendix. I would find it appropriate to have one page or so well referenced explanation of your theoretical model in the main text. Plos one does not have a word count limit. A figure of that framework that is more detailed than figure 1 or a summarizing table would be useful too.

**We revised the opening paragraph of Section 2.2 to clearly identify the two theoretical traditions (perceptions vs. technology–environment fit) and to frame Sections 2.2.1–2.2.3 as their systematic elaboration and integration. We clarified that some of the older references are foundational and remain critical to understanding the historical evolution of adoption frameworks. We also agree that more recent contributions can help illustrate the continuity of development, and we have included these as well. We believe these changes make the section clearer and situate the TAB framework more effectively. The relevant section of the text now reads:**

**The TAB framework builds on two established traditions in research on technology uptake: one emphasizing the role of user perceptions and the other emphasizing the fit between technology, tasks, and users. To make these links transparent, we organize this section into three parts. Section 2.2.1 outlines the importance of perceptions, drawing from models such as the Technology Acceptance Model (TAM) [31]. Section 2.2.2 describes the technology–environment fit tradition, including both early formulations [32,33] and more recent refinements [34]. Section 2.2.3 explains how TAB integrates these perspectives into a unified framework. Although some foundational contributions are older, they remain the basis for contemporary refinements, and we complement them with recent extensions [34] to ensure the framework reflects current scholarship**

3. Please be more clear that your main goal is or was to use the TAB framework in designing the interview guide and for analysis. For this a table or a figure of your model that is somehow consistent with the interview guide and the codebook (so far I can´t understand how fig. 1 and the interview guide are related to each others in the “headings” and “subheadings”) with its constructs would be very helpful. Otherwise it stands there and it is hard to understand. See how researchers use established frameworks, you are for sure aware of, e g https://implementationscience.biomedcentral.com/articles/10.1186/s13012-023-01296-x

**We agree that clearly indicating that using the TAB framework in designing the interview guide and for analysis is important. In section 2.1, we stated that “[t]he data collection and analysis will leverage the technological adoption behavior (TAB) framework.”**

**To improve clarity, we revised the column labels in Table 1 from “Heading” and “Subheading” to “TAB Construct” and “Associated Variable,” respectively, and added a note at the end of the table clarifying how these dimensions link directly to the TAB framework. This makes the relationship between the framework (Figure 1), the interview guide (Table 1), and the deductive codebook (Table 2) more explicit.**

3. Lines 167-169 How will physicians be recruited?

**We added specific details about our recruitment methods (emails, posted invitations, events, and personal contacts) in Section 2.3. The relevant text reads:**

**Recruitment will occur through organizational listserv emails, posted invitations on member websites, and outreach at educational events. In addition, physicians known to the research team may be approached via personal contacts; snowball sampling will be used when participants recommend colleagues.**

Minor points

1. Can you be more specific on what you mean for “disconfirming the evidence” (line 179)

**We clarified in Section 2.4 that “disconfirming evidence” refers to “actively probing for accounts that contradict or challenge emerging patterns or assumptions.”.**

2. I like how you engage in exploring the backgrounds of your team members. Please specify who is the main analyst who will undertake coding and development and refinement of teams. If you use parallel coding, please specify to what extend and why (best practice in qualitative research is to use it to incorporate different perspectives).

**We clarified who will conduct primary coding and explained that coding decisions will be refined collaboratively rather than through parallel coding (Section 2.5). The relevant section now reads:**

**Primary coding will be conducted by a new member of the research team after the person is trained in the TAB framework and qualitative coding. The person will be in charge of developing additional codes, and every code addition, removal, and refinement will be discussed with the senior author.**

3. Please be more specific on when you plan to use deductive and when you plan to use inductive coding (you call that elaborative coding, which is fine). Please also describe how you use your deductive and possibly inductive codes to elaborate themes. I don´t entirely understand how you could draw a thematic analysis from a code like “image” or “job relevance”, this needs some explanation.

**We use the codes to form analytic scaffold for organizing our data. The deductive codes draw from the TAB framework, and the inductive codes help capture ideas that may extend beyond the framework. By exploring how the codes—whether developed deductively or inductively—interact with each other, this can start to form higher order appreciation of the meaning behind the codes. The coalescing of such patterns can then guide the development of broader themes. The relevant sections of text include:**

**We aim to use both deductive and inductive coding approaches to flexibly understand and capture the content from the participants. Table 2 shows the series of deductive codes based on the TAB framework. The inductive coding refines the existing codes and / or develop new ones based on the empirically collected data [43]. We will rely on this approach when the data extend beyond the existing codes developed based on the TAB framework.**

**We will iteratively discuss within the group to distill the meaning of the findings, and as the relationships and dynamics of the codes emerge, this will lead us towards broader themes [44]. We aim to generate clear and concise definitions for each theme and review and refine the identified themes to ensure accurate representation.**

**For example, data may show that for physicians to consider an AI tool to be useful, it needs to help boost their “image” and be “relevant for their job.” Since a job may have fluctuating social definitions, this may interact with the code of image. Together, they might generate a theme about the interacting roles of these two dimensions for perceived usefulness. This example is a bit artificial, and we mainly worked with your comment to illustrate the process of how separate codes can facilitate the development of a theme. The actual analysis may identify different codes and themes depending on what the data reveals.**

4. Line 69-74: would phrase this with less emphasis as all the research published has several limitations and the research field is obviously quite new. Also, LLMs are known to have hallucinations that are unpredictable and, as far as I know, there are no validated LLMs that were proven safe and effective in substituting humans.

**We have** **de-emphasized the potential of current LLMs in contributing to diagnostic performance, and we also added more context with regard to novelty of the field and skepticism of early research. The relevant sections of the text now read:**

**In some cases, LLMs’ diagnostic performance may at times rival that of some human specialists [4–7]. These studies suggest promising performance in many contexts; however, this research is preliminary and subject to important limitations given the novelty of the field.**

5. Sentence on line 79 seems incomplete: “First, their roles cross both leadership and frontline service provision”…?

**Thank you.** **We looked over this sentence, and it has the necessary components of a complete sentence.**
