# Supplementary material for: Understanding primary care physicians’ perceptions of large language model adoption in clinical practice: A qualitative research protocol leveraging the technology adoption behavior framework
Source: PLoS One. 2026 Aug 7;21(8):e0355491. doi: 10.1371/journal.pone.0355491 (PMC13450787; doi:10.1371/journal.pone.0355491)
Supplement: Supplementary file 3 [file pone.0355491.s010.docx]

**Reviewer #3 Comments**

Thank you so much for this study protocol, addressing an important and actual topic in primary care.

**1)** First of all, an LLM is just a type of AI that has been trained to understand and generate human language, without any further definition of its application, interface or interacting target group.

Since each study participant brings different experiences and perspectives in the application of LLMs, the ideas about how an LLM might be used in daily medical practice will also vary greatly. Some may think of computer based Chatbots like ChatGPT, others might think of automated telefon calls or software applications to analyse text-based information. Some people might imagine automatic translating systems, others of tools for proper text summaries, program code generators, or software that might manage knowledge and big data.

To get results with real implications for primary health care, it would be very helpful to describe the participants before the interview what type of LLM they should imagine for which purpose, for which target groups(patients? physicians? non-physician GP practice staff?), which hardware will be used (phone, laptop, tablet, server) and whether it is intended for prevention, diagnostics or therapy.

**Thank you. We agree that different experiences and perspectives in the application of LLMs may drive varying perceptions of LLM usage. To address this, interviewers will briefly clarify the contexts under which the participants have interacted with LLMs. The interviewers will do so at the outset of each interview. The questions will ask the participant to clarify the examples of LLMs used or considered, intended purpose of such LLMs (e.g. prevention, diagnostics, or therapy), target user groups, potential hardware required, and relevant settings of use. We added the following to the Methodology section:

“Because different experiences and perspectives in the application of LLMs may drive varying perceptions of LLM usage, interviewers will briefly clarify the contexts under which the participants have interacted with LLMs. The interviewers will do so at the outset of each interview. The questions will ask the participant to clarify the examples of LLMs used or considered, intended purpose of such LLMs (e.g. prevention, diagnostics, or therapy), target user groups, potential hardware required, and relevant settings of use.”**

**We added the following interview questions:**

**In what context have you used, or do you imagine using, large language models in your practice? When you think about LLMs in your answers today, can you consider: a) What specific examples or types of tools are you primarily thinking of? B) are they used for diagnostics, prevention, or treatment? C) Who do you see as the main user of these tools? D) At a broad level, under what settings have you used or imagine using these tools in clinical practice? E) What types of hardware do you anticipate including (e.g. computer or smartphone)?**

**2)** The application of a theoretical model to structure data collection and analysis is highly recommended. However, I would have suggested to apply an already validated framework, instead of introducing a newly developed model. You might think of different possibilities, e.g. as the behavior change wheel, the consolidated framework for implementation research or the theoretical domains framework.

**Thank you for seeing value in using a theoretical framework to guide the data collection and analysis.**

**We followed the guidance from Varpio et al. in creating “a logically developed and connected set of concepts and premises—developed from one or more theories—that …scaffold a study ” [1]. The theoretical framework is a [tentative framework…proposed then refined as data are collected and as the researcher’s understanding evolves”** **[1].**

**To ensure the robustness of the framework, we first used validated frameworks to form the foundation of the framework. Appendix A describes the range of validated frameworks that we incorporated, which included the theory of reasoned action, the theory of planned behavior, technology acceptance model, fit among individuals, task, and technology model, and fit among individuals, skills, tasks, and technology model. Second, we leveraged empirical evidence to weave together the different validated frameworks. All of this is to ensure that the theoretical framework we lean on is as strong as possible for our research question.**

**We appreciated the recommendations to consider additional frameworks. However, the suggested frameworks appeared to draw from implementation science research. Implementation science is “the act of carrying an intention into effect” [2]. Applied to the context of AI, the implementation concept would prematurely assume that rolling out AI widely across clinical services is a worthwhile goal, and the research would then aim to identify what can facilitate such widespread uptake in a specific way.**

**We do not assume that increasing LLM uptake is worthwhile, and we do not seek to maximize doctors’ uptake of AI. For example, our research might capture how people may hold different views of what LLMs are and how they should be used. The participants’ responses might contradict the need to implement such tools. Because our assumption and goal of this research is exploratory, and because the project differs from the implementation science foci, we chose to maintain the current content of our theoretical framework without introducing additional elements from the suggested implementation science frameworks.**

**At the start of section 2.2 when we introduced the TAB theoretical framework, we added in the following text:**

**We followed the guidance from Varpio et al. in creating “a logically developed and connected set of concepts and premises—developed from one or more theories—that …scaffold a study” [1].**

3) It is a bit confusing to understand which methods had been already performed to develope the TAB modell ("camps"?) and which methods are part of the study protocoll.

I would recommend to clarify the above mentioned issues, to develope indicators that might be practically applicable in the improvement of primary health care.

**Thank you for highlighting this point. We followed guidance from Varpio et al. in developing a theoretical framework [1]. To clarify the distinction between framework development and the present study, we have explicitly stated that the TAB framework was developed by the authors through theoretical synthesis of existing adoption frameworks, as detailed in Appendix A, and is applied here to guide interview design and data analysis.**

**Reviewer #4 Comments**

The introduction is generally well written, but its organization could be improved for clarity and accessibility. Currently, it moves into discussing LLMs without first establishing the context or explaining what they are, which may confuse readers unfamiliar with the concept.

Consider starting with a brief definition of LLMs and why they are relevant to healthcare before introducing their potential applications. Additionally, clarify the scope of the discussion: Is the focus on LLMs for quality improvement, as day-to-day clinical assistants, or embedded within electronic medical records (EMRs)? These distinctions will help readers understand the purpose of the paper.

**Thank you for these helpful suggestions. To improve clarity and accessibility for readers unfamiliar with large language models, we added a brief definition of LLMs and their relevance to healthcare early in the introduction as follows:**

**“LLMs are a class of artificial intelligence systems trained on vast text datasets to understand and generate human-like language. In healthcare, their ability to process unstructured clinical information positions them as emerging tools for documentation, decision support, patient communication, and administrative workflows.”**

**We also clarified that rather than pre-specifying a single LLM application, interviewers will briefly elicit participants’ assumed context of LLM use at the outset of each interview and examine patterns across use-case strata during analysis.**

**We added the following interview questions to elicit participants’ assumed context of LLM use:**

**In what context have you used, or do you imagine using, LLMs in your practice? When you think about LLMs in your answers today, can you consider: a) What specific examples or types of tools are you primarily thinking of? B) are they used for diagnostics, prevention, or treatment? C) Who do you see as the main user of these tools? D) At a broad level, under what settings have you used or imagine using these tools in clinical practice? E) What types of hardware do you anticipate including (e.g. computer or smartphone)?**

**References Used in Reply to Reviewers**

1. **Varpio L, Paradis E, Uijtdehaage S, Young M. The distinctions between theory, theoretical framework, and conceptual framework. Acad Med. 2020;95(7):989-994.**
2. **Peters DH, Adam T, Alonge O, Agyepong IA, Tran N. Republished research: Implementation research: what it is and how to do it. Br J Sports Med. 2014;48(8):731-736.**
