# Supplementary material for: Understanding primary care physicians’ perceptions of large language model adoption in clinical practice: A qualitative research protocol leveraging the technology adoption behavior framework
Source: PLoS One. 2026 Aug 7;21(8):e0355491. doi: 10.1371/journal.pone.0355491 (PMC13450787; doi:10.1371/journal.pone.0355491)
Supplement: Peer review history

# Peer review: Decision Letter 0

**DOI:** 10.1371/journal.pone.0355491.r001

**Authors:** Ahmed Meri

3 Sep 2025

PONE-D-25-26184

Understanding Primary Care Physicians’ Perceptions of Large Language Model Adoption in Clinical Practice: A Qualitative Research Protocol Leveraging the Technology Adoption Behavior Framework

PLOS ONE

Dear Dr. Pokharel,

Thank you for submitting your manuscript to PLOS ONE. After careful consideration, we feel that it has merit but does not fully meet PLOS ONE’s publication criteria as it currently stands. Therefore, we invite you to submit a revised version of the manuscript that addresses the points raised during the review process.

Please submit your revised manuscript by Oct 18 2025 11:59PM. If you will need more time than this to complete your revisions, please reply to this message or contact the journal office at plosone@plos.org. When you're ready to submit your revision, log on to https://www.editorialmanager.com/pone/ and select the 'Submissions Needing Revision' folder to locate your manuscript file.

Please include the following items when submitting your revised manuscript:

- A rebuttal letter that responds to each point raised by the academic editor and reviewer(s). You should upload this letter as a separate file labeled 'Response to Reviewers'.
- A marked-up copy of your manuscript that highlights changes made to the original version. You should upload this as a separate file labeled 'Revised Manuscript with Track Changes'.
- An unmarked version of your revised paper without tracked changes. You should upload this as a separate file labeled 'Manuscript'.

If you would like to make changes to your financial disclosure, please include your updated statement in your cover letter. Guidelines for resubmitting your figure files are available below the reviewer comments at the end of this letter.

If applicable, we recommend that you deposit your laboratory protocols in protocols.io to enhance the reproducibility of your results. Protocols.io assigns your protocol its own identifier (DOI) so that it can be cited independently in the future. For instructions see: https://journals.plos.org/plosone/s/submission-guidelines#loc-laboratory-protocols. Additionally, PLOS ONE offers an option for publishing peer-reviewed Lab Protocol articles, which describe protocols hosted on protocols.io. Read more information on sharing protocols at https://plos.org/protocols?utm_medium=editorial-email&utm_source=authorletters&utm_campaign=protocols.

We look forward to receiving your revised manuscript.

Kind regards,

Ahmed Meri, Ph.D.

Academic Editor

PLOS ONE

Journal Requirements:

When submitting your revision, we need you to address these additional requirements.

1. Please ensure that your manuscript meets PLOS ONE's style requirements, including those for file naming. The PLOS ONE style templates can be found at

https://journals.plos.org/plosone/s/file?id=wjVg/PLOSOne_formatting_sample_main_body.pdf and

https://journals.plos.org/plosone/s/file?id=ba62/PLOSOne_formatting_sample_title_authors_affiliations.pdf

2. Please note that PLOS One has specific guidelines on code sharing for submissions in which author-generated code underpins the findings in the manuscript. In these cases, we expect all author-generated code to be made available without restrictions upon publication of the work. Please review our guidelines at https://journals.plos.org/plosone/s/materials-and-software-sharing#loc-sharing-code and ensure that your code is shared in a way that follows best practice and facilitates reproducibility and reuse.

3. Thank you for stating the following in the Competing Interests section:

“Dr. Sian Tsuei serves as a member on the Artificial Intelligence Advisory Council for the College of Canadian Family Physicians.”

Please confirm that this does not alter your adherence to all PLOS ONE policies on sharing data and materials, by including the following statement: "This does not alter our adherence to PLOS ONE policies on sharing data and materials.” (as detailed online in our guide for authors http://journals.plos.org/plosone/s/competing-interests). If there are restrictions on sharing of data and/or materials, please state these. Please note that we cannot proceed with consideration of your article until this information has been declared.

Please include your updated Competing Interests statement in your cover letter; we will change the online submission form on your behalf.

4. Your ethics statement should only appear in the Methods section of your manuscript. If your ethics statement is written in any section besides the Methods, please delete it from any other section.

5. If the reviewer comments include a recommendation to cite specific previously published works, please review and evaluate these publications to determine whether they are relevant and should be cited. There is no requirement to cite these works unless the editor has indicated otherwise.

[Note: HTML markup is below. Please do not edit.]

Reviewers' comments:

Reviewer's Responses to Questions

**Comments to the Author**

1. Does the manuscript provide a valid rationale for the proposed study, with clearly identified and justified research questions?

The research question outlined is expected to address a valid academic problem or topic and contribute to the base of knowledge in the field.

Reviewer #1: Yes

Reviewer #2: Yes

**********

2. Is the protocol technically sound and planned in a manner that will lead to a meaningful outcome and allow testing the stated hypotheses?

The manuscript should describe the methods in sufficient detail to prevent undisclosed flexibility in the experimental procedure or analysis pipeline, including sufficient outcome-neutral conditions (e.g. necessary controls, absence of floor or ceiling effects) to test the proposed hypotheses and a statistical power analysis where applicable. As there may be aspects of the methodology and analysis which can only be refined once the work is undertaken, authors should outline potential assumptions and explicitly describe what aspects of the proposed analyses, if any, are exploratory.

Reviewer #1: Yes

Reviewer #2: Partly

**********

3. Is the methodology feasible and described in sufficient detail to allow the work to be replicable?

Descriptions of methods and materials in the protocol should be reported in sufficient detail for another researcher to reproduce all experiments and analyses. The protocol should describe the appropriate controls, sample size calculations, and replication needed to ensure that the data are robust and reproducible.

Reviewer #1: Yes

Reviewer #2: No

**********

4. Have the authors described where all data underlying the findings will be made available when the study is complete?

The PLOS Data policy requires authors to make all data underlying the findings described in their manuscript fully available without restriction, with rare exception, at the time of publication. The data should be provided as part of the manuscript or its supporting information, or deposited to a public repository. For example, in addition to summary statistics, the data points behind means, medians and variance measures should be available. If there are restrictions on publicly sharing data—e.g. participant privacy or use of data from a third party—those must be specified.

Reviewer #1: Yes

Reviewer #2: Yes

**********

5. Is the manuscript presented in an intelligible fashion and written in standard English?

PLOS ONE does not copyedit accepted manuscripts, so the language in submitted articles must be clear, correct, and unambiguous. Any typographical or grammatical errors should be corrected at revision, so please note any specific errors here.

Reviewer #1: Yes

Reviewer #2: No

**********

6. Review Comments to the Author

Please use the space provided to explain your answers to the questions above and, if applicable, provide comments about issues authors must address before this protocol can be accepted for publication. You may also include additional comments for the author, including concerns about research or publication ethics.

You may also provide optional suggestions and comments to authors that they might find helpful in planning their study.

(Please upload your review as an attachment if it exceeds 20,000 characters)

Reviewer #1: PONE-D-25-26184

Understanding Primary Care Physicians’ Perceptions of Large Language Model Adoption in Clinical Practice: A Qualitative Research Protocol Leveraging the Technology Adoption Behavior Framework

Reviewer’s report

Dear Authors,

This study addresses a relevant and timely topic using a sound methodology. The manuscript is well written and thoughtfully prepared. Although I usually aim to provide critical and constructive feedback, I find this study protocol to be excellent. I have no concerns or further comments. I look forward to seeing the results of this study.

Reviewer #2: Thank you for allowing me to review this manuscript, which deals with a relevant topic, i.e. physicians´ perspectives of LLMs. The study design is appropriate to answer the research question.

I´d recommend the authors to revise the manuscript as follows:

- A summary of what previous studies found out on primary care physicians´ (or health professionals´) perspectives on large language models is missing. I agree that primary care physicians are those who do the heavy lifting, so understanding their perspectives is especially important. However, some studies, quantitative and qualitative, must exist out there, so it would be interesting to contextualize your study idea in the current scholarly discussion/state of the art.

- Para 2.2. is hard to follow. There is hardly any reference in the text but you seem to make a summary of what “experts” (who are they?) belive and did, you mention “camps” (what is that exactly?) without referencing any of your claims. References n. 28, 29, 30 are 17, 19 and 30 years old respectively, pretty outdated for a research protocol on LLMs.

It seems that the theoretical framework is your work, developed from previous studies that should be explained and referenced. Part of that is now in the appendix. I would find it appropriate to have one page or so well referenced explanation of your theoretical model in the main text. Plos one does not have a word count limit. A figure of that framework that is more detailed than figure 1 or a summarizing table would be useful too.

- Please be more clear that your main goal is or was to use the TAB framework in designing the interview guide and for analysis. For this a table or a figure of your model that is somehow consistent with the interview guide and the codebook (so far I can´t understand how fig. 1 and the interview guide are related to each others in the “headings” and “subheadings”) with its constructs would be very helpful. Otherwise it stands there and it is hard to understand. See how researchers use established frameworks, you are for sure aware of, e g https://implementationscience.biomedcentral.com/articles/10.1186/s13012-023-01296-x

- Lines 167-169: How? E-Mail, personal contacts? Invitation letters? Education events? Please be more specific.

- Can you be more specific on what you mean for “disconfirming the evidence” (line 179)

- I like how you engage in exploring the backgrounds of your team members. Please specify who is the main analyst who will undertake coding and development and refinement of teams. If you use parallel coding, please specify to what extend and why (best practice in qualitative research is to use it to incorporate different perspectives).

- Please be more specific on when you plan to use deductive and when you plan to use inductive coding (you call that elaborative coding, which is fine). Please also describe how you use your deductive and possibly inductive codes to elaborate themes. I don´t entirely understand how you could draw a thematic analysis from a code like “image” or “job relevance”, this needs some explanation.

Minor points

- Line 69-74: would phrase this with less emphasis as all the research published has several limitations and the research field is obviously quite new. Also, LLMs are known to have hallucinations that are unpredictable and, as far as I know, there are no validated LLMs that were proven safe and effective in substituting humans.

- Sentence on line 79 seems incomplete: “First, their roles cross both leadership and frontline service provision”…?

**********

7. PLOS authors have the option to publish the peer review history of their article (what does this mean?). If published, this will include your full peer review and any attached files.

If you choose “no”, your identity will remain anonymous but your review may still be made public.

**Do you want your identity to be public for this peer review?**  For information about this choice, including consent withdrawal, please see our Privacy Policy.

Reviewer #1: No

Reviewer #2: No

**********

[NOTE: If reviewer comments were submitted as an attachment file, they will be attached to this email and accessible via the submission site. Please log into your account, locate the manuscript record, and check for the action link "View Attachments". If this link does not appear, there are no attachment files.]

While revising your submission, please upload your figure files to the Preflight Analysis and Conversion Engine (PACE) digital diagnostic tool, https://pacev2.apexcovantage.com/. PACE helps ensure that figures meet PLOS requirements. To use PACE, you must first register as a user. Registration is free. Then, login and navigate to the UPLOAD tab, where you will find detailed instructions on how to use the tool. If you encounter any issues or have any questions when using PACE, please email PLOS at figures@plos.org. Please note that Supporting Information files do not need this step.

# Author comment: Author response to Decision Letter 1

**DOI:** 10.1371/journal.pone.0355491.r002

1 Oct 2025

Editor Comment 1:

Please ensure that your manuscript meets PLOS ONE's style requirements, including those for file naming. The PLOS ONE style templates can be found at

https://journals.plos.org/plosone/s/file?id=wjVg/PLOSOne_formatting_sample_main_body.pdf

and

https://journals.plos.org/plosone/s/file?id=ba62/PLOSOne_formatting_sample_title_authors_affiliations.pdf

Response:

Our team reviewed this again. To our knowledge, our format aligns with PLOS ONE’s style requirements.

Editor Comment 2:

Please note that PLOS One has specific guidelines on code sharing for submissions in which author-generated code underpins the findings in the manuscript. In these cases, we expect all author-generated code to be made available without restrictions upon publication of the work. Please review our guidelines at https://journals.plos.org/plosone/s/materials-and-software-sharing#loc-sharing-code

and ensure that your code is shared in a way that follows best practice and facilitates reproducibility and reuse.

Response:

This piece seems to refer to computational code instead of qualitative codes. We do not expect to use any computational code in our study, and we have explicitly described the deductive codes that we plan to use.

Editor Comment 3:

Thank you for stating the following in the Competing Interests section:

“Dr. Sian Tsuei serves as a member on the Artificial Intelligence Advisory Council for the College of Canadian Family Physicians.”

Please confirm that this does not alter your adherence to all PLOS ONE policies on sharing data and materials, by including the following statement: "This does not alter our adherence to PLOS ONE policies on sharing data and materials.” (as detailed online in our guide for authors http://journals.plos.org/plosone/s/competing-interests). If there are restrictions on sharing of data and/or materials, please state these. Please note that we cannot proceed with consideration of your article until this information has been declared.

Response:

We have now included in our manuscript the statement that "[t]his does not alter our adherence to PLOS ONE policies on sharing data and materials.”

Editor Comment 4:

Your ethics statement should only appear in the Methods section of your manuscript. If your ethics statement is written in any section besides the Methods, please delete it from any other section.

Response:

We have included the ethics statement in only the Methods. We removed the ethics statement from the Abstract section.

Editor Comment 5:

If the reviewer comments include a recommendation to cite specific previously published works, please review and evaluate these publications to determine whether they are relevant and should be cited. There is no requirement to cite these works unless the editor has indicated otherwise.

Response:

Thank you. The reviewers have not specifically asked us to cite any previously published work.

Reviewer 1 Comment:

Dear Authors,

This study addresses a relevant and timely topic using a sound methodology. The manuscript is well written and thoughtfully prepared. Although I usually aim to provide critical and constructive feedback, I find this study protocol to be excellent. I have no concerns or further comments. I look forward to seeing the results of this study.

Response:

Thank you for the kind comment. We appreciate that the work was of interest to you and met your standard. Please do look out for our subsequent work. We hope that it’ll continue to be of interest.

Reviewer 2 Comment 1:

A summary of what previous studies found out on primary care physicians´ (or health professionals´) perspectives on large language models is missing. I agree that primary care physicians are those who do the heavy lifting, so understanding their perspectives is especially important. However, some studies, quantitative and qualitative, must exist out there, so it would be interesting to contextualize your study idea in the current scholarly discussion/state of the art.

Response:

While previous work has examined general healthcare workers’ perception of LLMs [1], to our knowledge, assessment of clinician/healthcare worker perception of LLMs guided by theory is not present in the literature. The relevant sections of the text reads:

Although a small but growing body of work has begun to examine clinicians’ perceptions of LLMs [17–19], prior studies are largely atheoretical or lightly theory informed and rarely focus on primary care. To our knowledge, no study in primary care has used an explicit framework to guide both data collection and analysis.

Reviewer 2 Comment 2:

Para 2.2. is hard to follow. There is hardly any reference in the text but you seem to make a summary of what “experts” (who are they?) belive and did, you mention “camps” (what is that exactly?) without referencing any of your claims. References n. 28, 29, 30 are 17, 19 and 30 years old respectively, pretty outdated for a research protocol on LLMs.

It seems that the theoretical framework is your work, developed from previous studies that should be explained and referenced. Part of that is now in the appendix. I would find it appropriate to have one page or so well referenced explanation of your theoretical model in the main text. Plos one does not have a word count limit. A figure of that framework that is more detailed than figure 1 or a summarizing table would be useful too.

Response:

We revised the opening paragraph of Section 2.2 to clearly identify the two theoretical traditions (perceptions vs. technology–environment fit) and to frame Sections 2.2.1–2.2.3 as their systematic elaboration and integration. We clarified that some of the older references are foundational and remain critical to understanding the historical evolution of adoption frameworks. We also agree that more recent contributions can help illustrate the continuity of development, and we have included these as well. We believe these changes make the section clearer and situate the TAB framework more effectively. The relevant section of the text now reads:

The TAB framework builds on two established traditions in research on technology uptake: one emphasizing the role of user perceptions and the other emphasizing the fit between technology, tasks, and users. To make these links transparent, we organize this section into three parts. Section 2.2.1 outlines the importance of perceptions, drawing from models such as the Technology Acceptance Model (TAM) [31]. Section 2.2.2 describes the technology–environment fit tradition, including both early formulations [32,33] and more recent refinements [34]. Section 2.2.3 explains how TAB integrates these perspectives into a unified framework. Although some foundational contributions are older, they remain the basis for contemporary refinements, and we complement them with recent extensions [34] to ensure the framework reflects current scholarship.

Reviewer 2 Comment 3:

Please be more clear that your main goal is or was to use the TAB framework in designing the interview guide and for analysis. For this a table or a figure of your model that is somehow consistent with the interview guide and the codebook (so far I can´t understand how fig. 1 and the interview guide are related to each others in the “headings” and “subheadings”) with its constructs would be very helpful. Otherwise it stands there and it is hard to understand. See how researchers use established frameworks, you are for sure aware of, e g https://implementationscience.biomedcentral.com/articles/10.1186/s13012-023-01296-x

Response:

We agree that clearly indicating that using the TAB framework in designing the interview guide and for analysis is important. In section 2.1, we stated that “[t]he data collection and analysis will leverage the technological adoption behavior (TAB) framework.” To improve clarity, we revised the column labels in Table 1 from “Heading” and “Subheading” to “TAB Construct” and “Associated Variable,” respectively, and added a note at the end of the table clarifying how these dimensions link directly to the TAB framework. This makes the relationship between the framework (Figure 1), the interview guide (Table 1), and the deductive codebook (Table 2) more explicit.

Reviewer 2 Comment 4:

Lines 167-169 How will physicians be recruited?

Response:

We added specific details about our recruitment methods (emails, posted invitations, events, and personal contacts) in Section 2.3. The relevant text reads: Recruitment will occur through organizational listserv emails, posted invitations on member websites, and outreach at educational events. In addition, physicians known to the research team may be approached via personal contacts; snowball sampling will be used when participants recommend colleagues.

Reviewer 2 Minor Comment 1:

Can you be more specific on what you mean for “disconfirming the evidence” (line 179)

Response:

We clarified in Section 2.4 that “disconfirming evidence” refers to “actively probing for accounts that contradict or challenge emerging patterns or assumptions.”

Reviewer 2 Minor Comment 2:

I like how you engage in exploring the backgrounds of your team members. Please specify who is the main analyst who will undertake coding and development and refinement of teams. If you use parallel coding, please specify to what extend and why (best practice in qualitative research is to use it to incorporate different perspectives).

Response:

We clarified who will conduct primary coding and explained that coding decisions will be refined collaboratively rather than through parallel coding (Section 2.5). The relevant section now reads: Primary coding will be conducted by a new member of the research team after the person is trained in the TAB framework and qualitative coding. The person will be in charge of developing additional codes, and every code addition, removal, and refinement will be discussed with the senior author.

Reviewer 2 Minor Comment 3:

Please be more specific on when you plan to use deductive and when you plan to use inductive coding (you call that elaborative coding, which is fine). Please also describe how you use your deductive and possibly inductive codes to elaborate themes. I don´t entirely understand how you could draw a thematic analysis from a code like “image” or “job relevance”, this needs some explanation.

Response:

We use the codes to form analytic scaffold for organizing our data. The deductive codes draw from the TAB framework, and the inductive codes help capture ideas that may extend beyond the framework. By exploring how the codes—whether developed deductively or inductively—interact with each other, this can start to form higher order appreciation of the meaning behind the codes. The coalescing of such patterns can then guide the development of broader themes. The relevant sections of text include: We aim to use both deductive and inductive coding approaches to flexibly understand and capture the content from the participants. Table 2 shows the series of deductive codes based on the TAB framework. The inductive coding refines the existing codes and / or develop new ones based on the empirically collected data [43]. We will rely on this approach when the data extend beyond the existing codes developed based on the TAB framework. We will iteratively discuss within the group to distill the meaning of the findings, and as the relationships and dynamics of the codes emerge, this will lead us towards broader themes [44]. We aim to generate clear and concise definitions for each theme and review and refine the identified themes to ensure accurate representation. For example, data may show that for physicians to consider an AI tool to be useful, it needs to help boost their “image” and be “relevant for their job.” Since a job may have fluctuating social definitions, this may interact with the code of image. Together, they might generate a theme about the interacting roles of these two dimensions for perceived usefulness. This example is a bit artificial, and we mainly worked with your comment to illustrate the process of how separate codes can facilitate the development of a theme. The actual analysis may identify different codes and themes depending on what the data reveals.

Reviewer 2 Minor Comment 4:

Line 69-74: would phrase this with less emphasis as all the research published has several limitations and the research field is obviously quite new. Also, LLMs are known to have hallucinations that are unpredictable and, as far as I know, there are no validated LLMs that were proven safe and effective in substituting humans.

Response:

We have de-emphasized the potential of current LLMs in contributing to diagnostic performance, and we also added more context with regard to novelty of the field and skepticism of early research. The relevant sections of the text now read: In some cases, LLMs’ diagnostic performance may at times rival that of some human specialists [4–7]. These studies suggest promising performance in many contexts; however, this research is preliminary and subject to important limitations given the novelty of the field.

Reviewer 2 Minor Comment 5:

Sentence on line 79 seems incomplete: “First, their roles cross both leadership and frontline service provision”…?

Response:

Thank you. We looked over this sentence, and it has the necessary components of a complete sentence.

Attachment Submitted filename: Response to Reviewers.docx

# Peer review: Decision Letter 1

**DOI:** 10.1371/journal.pone.0355491.r003

**Authors:** Ahmed Meri

8 Dec 2025

PONE-D-25-26184R1

Understanding Primary Care Physicians’ Perceptions of Large Language Model Adoption in Clinical Practice: A Qualitative Research Protocol Leveraging the Technology Adoption Behavior Framework

PLOS One

Dear Dr. Pokharel,

Thank you for submitting your manuscript to PLOS ONE. After careful consideration, we feel that it has merit but does not fully meet PLOS ONE’s publication criteria as it currently stands. Therefore, we invite you to submit a revised version of the manuscript that addresses the points raised during the review process.

Please submit your revised manuscript by Jan 22 2026 11:59PM. If you will need more time than this to complete your revisions, please reply to this message or contact the journal office at plosone@plos.org. When you're ready to submit your revision, log on to https://www.editorialmanager.com/pone/ and select the 'Submissions Needing Revision' folder to locate your manuscript file.

Please include the following items when submitting your revised manuscript:

- A rebuttal letter that responds to each point raised by the academic editor and reviewer(s). You should upload this letter as a separate file labeled 'Response to Reviewers'.
- A marked-up copy of your manuscript that highlights changes made to the original version. You should upload this as a separate file labeled 'Revised Manuscript with Track Changes'.
- An unmarked version of your revised paper without tracked changes. You should upload this as a separate file labeled 'Manuscript'.

If you would like to make changes to your financial disclosure, please include your updated statement in your cover letter. Guidelines for resubmitting your figure files are available below the reviewer comments at the end of this letter.

If applicable, we recommend that you deposit your laboratory protocols in protocols.io to enhance the reproducibility of your results. Protocols.io assigns your protocol its own identifier (DOI) so that it can be cited independently in the future. For instructions see: https://journals.plos.org/plosone/s/submission-guidelines#loc-laboratory-protocols. Additionally, PLOS ONE offers an option for publishing peer-reviewed Lab Protocol articles, which describe protocols hosted on protocols.io. Read more information on sharing protocols at https://plos.org/protocols?utm_medium=editorial-email&utm_source=authorletters&utm_campaign=protocols.

We look forward to receiving your revised manuscript.

Kind regards,

Ahmed Meri, Ph.D.

Academic Editor

PLOS One

Journal Requirements:

If the reviewer comments include a recommendation to cite specific previously published works, please review and evaluate these publications to determine whether they are relevant and should be cited. There is no requirement to cite these works unless the editor has indicated otherwise.

[Note: HTML markup is below. Please do not edit.]

Reviewers' comments:

Reviewer's Responses to Questions

**Comments to the Author**

1. Does the manuscript provide a valid rationale for the proposed study, with clearly identified and justified research questions?

The research question outlined is expected to address a valid academic problem or topic and contribute to the base of knowledge in the field.

Reviewer #3: Yes

Reviewer #4: Yes

**********

2. Is the protocol technically sound and planned in a manner that will lead to a meaningful outcome and allow testing the stated hypotheses?

The manuscript should describe the methods in sufficient detail to prevent undisclosed flexibility in the experimental procedure or analysis pipeline, including sufficient outcome-neutral conditions (e.g. necessary controls, absence of floor or ceiling effects) to test the proposed hypotheses and a statistical power analysis where applicable. As there may be aspects of the methodology and analysis which can only be refined once the work is undertaken, authors should outline potential assumptions and explicitly describe what aspects of the proposed analyses, if any, are exploratory.

Reviewer #3: No

Reviewer #4: Partly

**********

3. Is the methodology feasible and described in sufficient detail to allow the work to be replicable?

Descriptions of methods and materials in the protocol should be reported in sufficient detail for another researcher to reproduce all experiments and analyses. The protocol should describe the appropriate controls, sample size calculations, and replication needed to ensure that the data are robust and reproducible.

Reviewer #3: Yes

Reviewer #4: Yes

**********

4. Have the authors described where all data underlying the findings will be made available when the study is complete?

The PLOS Data policy requires authors to make all data underlying the findings described in their manuscript fully available without restriction, with rare exception, at the time of publication. The data should be provided as part of the manuscript or its supporting information, or deposited to a public repository. For example, in addition to summary statistics, the data points behind means, medians and variance measures should be available. If there are restrictions on publicly sharing data—e.g. participant privacy or use of data from a third party—those must be specified.

Reviewer #3: Yes

Reviewer #4: Yes

**********

5. Is the manuscript presented in an intelligible fashion and written in standard English?

PLOS ONE does not copyedit accepted manuscripts, so the language in submitted articles must be clear, correct, and unambiguous. Any typographical or grammatical errors should be corrected at revision, so please note any specific errors here.

Reviewer #3: Yes

Reviewer #4: Yes

**********

6. Review Comments to the Author

Please use the space provided to explain your answers to the questions above and, if applicable, provide comments about issues authors must address before this protocol can be accepted for publication. You may also include additional comments for the author, including concerns about research or publication ethics.

You may also provide optional suggestions and comments to authors that they might find helpful in planning their study.

(Please upload your review as an attachment if it exceeds 20,000 characters)

Reviewer #3: Thank you so much for this study protocol, adressing an important and actual topic in primary care.

However, I have some concerns that might have to be addressed before data collection.

1) First of all, an LLM is just a type of AI that has been trained to understand and generate human language, without any further definition of its application, interface or interacting targetgroup.

Since each study participant brings different experiences and perspectives in the application of LLMs, the ideas about how an LLM might be used in daily medical practice will also vary greatly. Some may think of computerbased Chatbots like ChtGPT, others might think of automated telefon calls or software applications to analyse textbased information. Some people might imagine automatic translating systems, others of tools for proper text summaries, program code generaters, or software that might manage knowledge and big data.

To get results with real implications for primary health care, it would be very helpful to describe the participants before the interview what type of LLM they should imagine for which purpose, for which targetgroupt (patients? physicians? non-physician GP practice staff?), which hardware will be used (phone, laptop, tablet, server) and whether it is intended for prevention, diagnostics or therapy.

2) The application of a theoretic modell to structure data collection and analysis is highly recommended. However, I would have suggested to apply an already validated framework, instead of introducing a newly developed model. You might think of different possibilites, e.g. as the behavior change wheel, the consolidated framework for implementation research or the theoretical domains framework.

3) It is a bit confusing to understand wich methods had been already performed to develope the TAB modell ("camps"?) and which methods are part of the study protocoll.

I would recommend to clarify the above mentioned issues, to develope indicators that might be practically applicable in the improvement of primary health care.

Best wishes

Reviewer #4: The introduction is generally well written, but its organization could be improved for clarity and accessibility. Currently, it moves into discussing LLMs without first establishing the context or explaining what they are, which may confuse readers unfamiliar with the concept.

Consider starting with a brief definition of LLMs and why they are relevant to healthcare before introducing their potential applications. Additionally, clarify the scope of the discussion: Is the focus on LLMs for quality improvement, as day-to-day clinical assistants, or embedded within electronic medical records (EMRs)? These distinctions will help readers understand the purpose of the paper.

**********

7. PLOS authors have the option to publish the peer review history of their article (what does this mean?). If published, this will include your full peer review and any attached files.

If you choose “no”, your identity will remain anonymous but your review may still be made public.

**Do you want your identity to be public for this peer review?**  For information about this choice, including consent withdrawal, please see our Privacy Policy.

Reviewer #3: **Yes:** Linda Sanftenberg

Reviewer #4: No

**********

[NOTE: If reviewer comments were submitted as an attachment file, they will be attached to this email and accessible via the submission site. Please log into your account, locate the manuscript record, and check for the action link "View Attachments". If this link does not appear, there are no attachment files.]

To ensure your figures meet our technical requirements, please review our figure guidelines: https://journals.plos.org/plosone/s/figures

You may also use PLOS’s free figure tool, NAAS, to help you prepare publication quality figures: https://journals.plos.org/plosone/s/figures#loc-tools-for-figure-preparation.

NAAS will assess whether your figures meet our technical requirements by comparing each figure against our figure specifications.

# Author comment: Author response to Decision Letter 2

**DOI:** 10.1371/journal.pone.0355491.r004

15 Feb 2026

Reviewer 3 Comment 1:

First of all, an LLM is just a type of AI that has been trained to understand and generate human language, without any further definition of its application, interface or interacting target group.

Since each study participant brings different experiences and perspectives in the application of LLMs, the ideas about how an LLM might be used in daily medical practice will also vary greatly. Some may think of computer based Chatbots like ChatGPT, others might think of automated telefon calls or software applications to analyse text-based information. Some people might imagine automatic translating systems, others of tools for proper text summaries, program code generators, or software that might manage knowledge and big data.

To get results with real implications for primary health care, it would be very helpful to describe the participants before the interview what type of LLM they should imagine for which purpose, for which target groups(patients? physicians? non-physician GP practice staff?), which hardware will be used (phone, laptop, tablet, server) and whether it is intended for prevention, diagnostics or therapy.

Response:

Thank you. We agree that different experiences and perspectives in the application of LLMs may drive varying perceptions of LLM usage. To address this, interviewers will briefly clarify the contexts under which the participants have interacted with LLMs. The interviewers will do so at the outset of each interview. The questions will ask the participant to clarify the examples of LLMs used or considered, intended purpose of such LLMs (e.g. prevention, diagnostics, or therapy), target user groups, potential hardware required, and relevant settings of use. We added the following to the Methodology section:

“Because different experiences and perspectives in the application of LLMs may drive varying perceptions of LLM usage, interviewers will briefly clarify the contexts under which the participants have interacted with LLMs. The interviewers will do so at the outset of each interview. The questions will ask the participant to clarify the examples of LLMs used or considered, intended purpose of such LLMs (e.g. prevention, diagnostics, or therapy), target user groups, potential hardware required, and relevant settings of use.”

We added the following interview questions:

In what context have you used, or do you imagine using, LLMs in your practice? When you think about LLMs in your answers today, can you consider: a) What specific examples or types of tools are you primarily thinking of? B) are they used for diagnostics, prevention, or treatment? C) Who do you see as the main user of these tools? D) At a broad level, under what settings have you used or imagine using these tools in clinical practice? E) What types of hardware do you anticipate including (e.g. computer or smartphone)?

Reviewer 3 Comment 2:

The application of a theoretical model to structure data collection and analysis is highly recommended. However, I would have suggested to apply an already validated framework, instead of introducing a newly developed model. You might think of different possibilities, e.g. as the behavior change wheel, the consolidated framework for implementation research or the theoretical domains framework.

Response:

Thank you for seeing value in using a theoretical framework to guide the data collection and analysis.

We followed the guidance from Varpio et al. in creating “a logically developed and connected set of concepts and premises—developed from one or more theories—that …scaffold a study ” [1]. The theoretical framework is a [tentative framework…proposed then refined as data are collected and as the researcher’s understanding evolves” [1].

To ensure the robustness of the framework, we first used validated frameworks to form the foundation of the framework. Appendix A describes the range of validated frameworks that we incorporated, which included the theory of reasoned action, the theory of planned behavior, technology acceptance model, fit among individuals, task, and technology model, and fit among individuals, skills, tasks, and technology model. Second, we leveraged empirical evidence to weave together the different validated frameworks. All of this is to ensure that the theoretical framework we lean on is as strong as possible for our research question.

We appreciated the recommendations to consider additional frameworks. However, the suggested frameworks appeared to draw from implementation science research. Implementation science is “the act of carrying an intention into effect” [2]. Applied to the context of AI, the implementation concept would prematurely assume that rolling out AI widely across clinical services is a worthwhile goal, and the research would then aim to identify what can facilitate such widespread uptake in a specific way.

We do not assume that increasing LLM uptake is worthwhile, and we do not seek to maximize doctors’ uptake of AI. For example, our research might capture how people may hold different views of what LLMs are and how they should be used. The participants’ responses might contradict the need to implement such tools. Because our assumption and goal of this research is exploratory, and because the project differs from the implementation science foci, we chose to maintain the current content of our theoretical framework without introducing additional elements from the suggested implementation science frameworks.

At the start of section 2.2 when we introduced the TAB theoretical framework, we added in the following text:

We followed the guidance from Varpio et al. in creating “a logically developed and connected set of concepts and premises—developed from one or more theories—that …scaffold a study” [1].

Reviewer 3 Comment 3:

It is a bit confusing to understand which methods had been already performed to develop the TAB model ("camps"?) and which methods are part of the study protocol.

I would recommend to clarify the above mentioned issues, to develop indicators that might be practically applicable in the improvement of primary health care.

Response:

Thank you for highlighting this point. We followed guidance from Varpio et al. in developing a theoretical framework [1].To clarify the distinction between framework development and the present study, we have explicitly stated that the TAB framework was developed by the authors through theoretical synthesis of existing adoption frameworks, as detailed in Appendix A, and is applied here to guide interview design and data analysis.

Reviewer 4 Comment 1:

The introduction is generally well written, but its organization could be improved for clarity and accessibility. Currently, it moves into discussing LLMs without first establishing the context or explaining what they are, which may confuse readers unfamiliar with the concept.

Consider starting with a brief definition of LLMs and why they are relevant to healthcare before introducing their potential applications. Additionally, clarify the scope of the discussion: Is the focus on LLMs for quality improvement, as day-to-day clinical assistants, or embedded within electronic medical records (EMRs)? These distinctions will help readers understand the purpose of the paper.

Response:

Thank you for these helpful suggestions. To improve clarity and accessibility for readers unfamiliar with large language models, we added a brief definition of LLMs and their relevance to healthcare early in the introduction as follows:

“LLMs are a class of artificial intelligence systems trained on vast text datasets to understand and generate human-like language. In healthcare, their ability to process unstructured clinical information positions them as emerging tools for documentation, decision support, patient communication, and administrative workflows.”

We also clarified that rather than pre-specifying a single LLM application, interviewers will briefly elicit participants’ assumed context of LLM use at the outset of each interview and examine patterns across use-case strata during analysis.

We added the following interview questions to elicit participants’ assumed context of LLM use:

In what context have you used, or do you imagine using, LLMs in your practice? When you think about LLMs in your answers today, can you consider: a) What specific examples or types of tools are you primarily thinking of? B) are they used for diagnostics, prevention, or treatment? C) Who do you see as the main user of these tools? D) At a broad level, under what settings have you used or imagine using these tools in clinical practice? E) What types of hardware do you anticipate including (e.g. computer or smartphone)?

References Used in Reply to Reviewers:

1. Varpio L, Paradis E, Uijtdehaage S, Young M. The distinctions between theory, theoretical framework, and conceptual framework. Acad Med. 2020;95(7):989-994.

2.Peters DH, Adam T, Alonge O, Agyepong IA, Tran N. Republished research: Implementation research: what it is and how to do it. Br J Sports Med. 2014;48(8):731-736.

Attachment Submitted filename: Response to Reviewers February 2026.docx

# Editor report: Decision Letter 2

**DOI:** 10.1371/journal.pone.0355491.r005

**Authors:** Noah Hammarlund

22 Jul 2026

Understanding Primary Care Physicians’ Perceptions of Large Language Model Adoption in Clinical Practice: A Qualitative Research Protocol Leveraging the Technology Adoption Behavior Framework

PONE-D-25-26184R2

Dear Dr. Pokharel,

We’re pleased to inform you that your manuscript has been judged scientifically suitable for publication and will be formally accepted for publication once it meets all outstanding technical requirements.

Within one week, you’ll receive an e-mail detailing the required amendments. When these have been addressed, you’ll receive a formal acceptance letter and your manuscript will be scheduled for publication.

An invoice will be generated when your article is formally accepted. Please note, if your institution has a publishing partnership with PLOS and your article meets the relevant criteria, all or part of your publication costs will be covered. Please make sure your user information is up-to-date by logging into Editorial Manager at Editorial Manager® and clicking the ‘Update My Information' link at the top of the page. For questions related to billing, please contact billing support.

If your institution or institutions have a press office, please notify them about your upcoming paper to help maximize its impact. If they’ll be preparing press materials, please inform our press team as soon as possible -- no later than 48 hours after receiving the formal acceptance. Your manuscript will remain under strict press embargo until 2 pm Eastern Time on the date of publication. For more information, please contact onepress@plos.org.

Kind regards,

Noah Hammarlund

Academic Editor

PLOS One

Additional Editor Comments (optional):

Reviewers' comments:

# Editor report: Acceptance letter

**DOI:** 10.1371/journal.pone.0355491.r006

**Authors:** Noah Hammarlund

PONE-D-25-26184R2

PLOS One

Dear Dr. Pokharel,

I'm pleased to inform you that your manuscript has been deemed suitable for publication in PLOS One. Congratulations! Your manuscript is now being handed over to our production team.

At this stage, our production department will prepare your paper for publication. This includes ensuring the following:

* All references, tables, and figures are properly cited

* All relevant supporting information is included in the manuscript submission,

* There are no issues that prevent the paper from being properly typeset

You will receive further instructions from the production team, including instructions on how to review your proof when it is ready. Please keep in mind that we are working through a large volume of accepted articles, so please give us a few days to review your paper and let you know the next and final steps.

Lastly, if your institution or institutions have a press office, please let them know about your upcoming paper now to help maximize its impact. If they'll be preparing press materials, please inform our press team within the next 48 hours. Your manuscript will remain under strict press embargo until 2 pm Eastern Time on the date of publication. For more information, please contact onepress@plos.org.

You will receive an invoice from PLOS for your publication fee after your manuscript has reached the completed accept phase. If you receive an email requesting payment before acceptance or for any other service, this may be a phishing scheme. Learn how to identify phishing emails and protect your accounts at https://explore.plos.org/phishing.

If we can help with anything else, please email us at customercare@plos.org.

Thank you for submitting your work to PLOS ONE and supporting open access.

Kind regards,

PLOS ONE Editorial Office Staff

on behalf of

Dr. Noah Hammarlund

Academic Editor

PLOS One
